# Intratumoral microbiota: bidirectional modulation of tumor radiosensitivity

**DOI:** 10.3389/fimmu.2026.1945760

**Published:** 2026-10-01

**Authors:** Fangfei Zhu, Shuting Han, Wenxiao Jia, Yiming Cao, Hui Zhu

**Affiliations:** 1 Clinical Oncology College, Shandong Cancer Hospital and Institute, Shandong First Medical University & Shandong Academy of Medical Sciences, Jinan, Shandong, China; 2 Department of Radiation Oncology, Shandong Cancer Hospital and Institute, Shandong First Medical University & Shandong Academy of Medical Sciences, Jinan, Shandong, China; 3 Department of Oncology, Xintai Municipal Hospital of Traditional Chinese Medicine, Xintai, Shandong, China

**Keywords:** cancer, DNA damage and repair, intratumoral microbiota, radiosensitivity, radiotherapy

## Abstract

The term “intratumoral microbiota” refers to the microbial communities that reside within tumor tissues, including bacteria, fungi, mycoplasma, and viruses. Accumulating evidence indicates that the intratumoral microbiota is closely associated with tumor initiation and progression. Nevertheless, the impact of these microbes on radiotherapy outcomes remains largely unexplored. In this review, we discussed how the intratumoral microbiota influences radiation response, highlighting the diverse mechanisms through which microbial communities modulate tumor cell behavior. Specifically, intratumoral microbes may confer radioresistance by enhancing DNA damage repair, reprogramming core metabolic pathways, regulating the cell cycle, and modulating autophagy, thereby diminishing radiotherapy efficacy. Conversely, certain microorganisms can enhance radiosensitivity by directly inducing DNA damage in host tumor cells, activating antitumor immune responses, or elevating intracellular reactive oxygen species(ROS) levels, thereby amplifying radiation-induced cytotoxicity. Additionally, microbial aggravation of tumor hypoxia may further diminish radiosensitivity by creating a more radioresistant tumor microenvironment. Therefore, the regulatory role of the intratumoral microbiota in radiotherapy sensitivity provides a novel direction for cancer therapy. Strategies that precisely target the intratumoral microbiota hold promise as important approaches for overcoming radioresistance and improving treatment outcomes in patients with cancer.

## 1 Introduction

The human body harbors a vast number of microorganisms comparable to human cells (1), some of which contribute to tumorigenesis via direct carcinogenesis, chronic inflammation, or immune evasion (2). For example, high-risk human papillomavirus (HPV)is linked to cervical, anal, and certain oropharyngeal cancers (3); Hepatitis B virus (HBV) and Hepatitis C virus (HCV) increase hepatocellular carcinoma risk (4, 5); Epstein–Barr virus (EBV) is associated with Burkitt lymphoma, nasopharyngeal carcinoma, Hodgkin lymphoma, and certain gastric cancers (6, 7). Among bacteria, *Helicobacter pylori* promotes gastric adenocarcinoma and mucosa-associated lymphoid tissue lymphoma via chronic gastritis, epithelial damage, and virulence factors such as CagA (8). With advancements in metagenomics and imaging technologies, the research focus is shifting from the role of the intratumoral microbiota in tumor initiation and progression to its role in the tumor treatment response. The concept of the intratumoral microbiota refers to the dynamic community within tumor tissues composed of bacteria, viruses, fungi, and mycoplasma. In the 19th century, Robert Koch and Louis Pasteur made initial observations of bacteria within tumor sites (9). In 1983, Marshall and Warren cultured *Helicobacter pylori*, further establishing its role in the etiology of gastric cancer (10, 11). In 2020, Nejman et al. conducted the first systematic study of the microbiome across multiple tumor types. Using 16S rRNA sequencing and Fluorescence *in situ* hybridization (FISH), they not only confirmed the widespread presence of microbes in the tumor microenvironment. but also showed that microbes can colonize both cancer cells and immune cells (12).

Multiple studies have shown that the intratumoral microbiota can significantly interfere with the efficacy of immune therapy and chemotherapy. In the context of immunotherapy, in a melanoma model, intratumoral *Lactobacillus reuteri* continuously releases dietary tryptophan metabolites, such as Indole-3-aldehyde, which activate the Aryl Hydrocarbon Receptor signaling pathway in CD8^+^ T cells, promoting interferon-gamma (IFN-γ) secretion and significantly increasing the efficacy of anti-programmed death-ligand 1(anti-PD-L1) therapy (13). Butyrate produced by intratumoral anaerobic bacteria has been reported to promote an anti-inflammatory (M2-like) macrophage phenotype and regulatory T-cell differentiation in gut-microbiota studies (14); whether such effects enhance M2 macrophage polarization, inhibit CD8^+^ T-cell infiltration, or compromise anti-PD-1 therapy in the tumor microenvironment warrants direct investigation. In the field of chemotherapy, a summary of data from 16 clinical cohorts indicated that patients with intratumoral enrichment of *Akkermansia* or *Bifidobacterium* presented a 20%–25% increase in the disease control rate after first-line chemotherapy, along with a reduction in immune-related adverse events (15). In pancreatic ductal adenocarcinoma(PDAC), *Bacteroides* can secrete long-chain cytidine deaminase, which metabolizes gemcitabine into an inactive product. However, after bacterial depletion with ciprofloxacin, the intratumoral concentration of the active drug increased 2.4-fold, and tumor growth was delayed by 45% (16). These findings indicate that the elimination of the intratumoral microbiota can restore chemotherapy sensitivity (17).

Radiotherapy uses high-energy ionizing radiation, including X-rays, γ-rays, high-energy electrons, and heavy ions, to induce DNA damage in cancer cells. This damage inhibits tumor-cell proliferation and ultimately contributes to tumor cell death. This modality can be used as a curative treatment or in combination with surgery, chemotherapy, or immunotherapy, and currently constitutes a central component of therapy for approximately 50% of cancer patients at some point during their disease course (18). Tumor radiosensitivity, a key factor influencing therapeutic outcomes and patient survival, is associated with DNA damage, DNA damage repair capacity, cell cycle arrest, autophagy regulation, cancer stem cell presence, and tumor cell metabolism (19, 20). Accumulating evidence suggests that the intratumoral microbiota is not only widely present in various solid tumors but also influences tumor cell responses to radiation through multiple mechanisms (21). For example, microbial metabolites or microbial regulation of tumor-cell signaling can promote apoptosis or senescence, thereby enhancing radiosensitivity (21). Conversely, certain microbes may weaken the cytotoxic effects of radiation and induce radioresistance by promoting DNA repair in tumor cells, regulating antioxidant stress responses, or remodeling the tumor vasculature (22). These complex and diverse regulatory mechanisms ultimately lead to different radiotherapy responses and clinical outcomes, highlighting the significant role of the intratumoral microbiota in regulating radiotherapy sensitivity. This review explores the multifaceted effects of the intratumoral microbiota on radiotherapy sensitivity, providing a biological rationale for microbiota-targeted strategies to overcome radioresistance and improve patient outcomes. As summarized in **Figure 1**, the regulatory impact of the intratumoral microbiota on radiosensitivity is bidirectional: certain microbial taxa potentiate radiation-induced cytotoxicity (radiosensitization), while others activate pro-survival pathways that confer therapeutic resistance (radioresistance). The following sections dissect these opposing mechanisms across key biological processes, including DNA damage repair, metabolic reprogramming, immune modulation, and tumor microenvironment remodeling.

**Figure 1:**
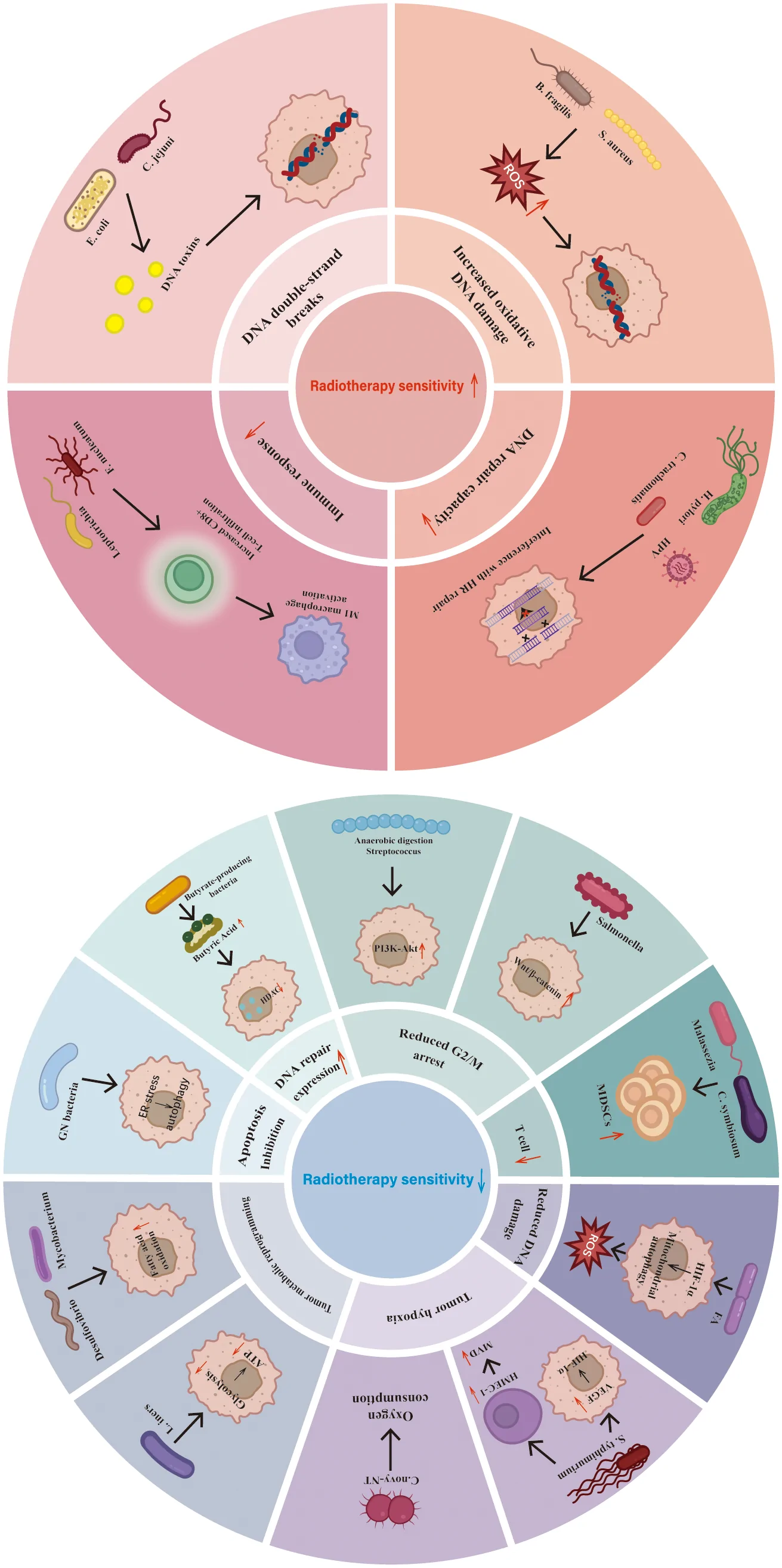
Bidirectional regulation of tumor radiosensitivity by the intratumoral microbiota. This schematic illustrates the dual roles of intratumoral microorganisms in modulating the response to radiotherapy, categorized into radiosensitizing (top panel, red arrows) and radioresistance (bottom panel, blue arrows) mechanisms. Top panel (Radiosensitization): Selected microbes enhance the efficacy of radiotherapy through the following pathways: (1) Direct DNA damage: Toxins such as colibactin (pks^+^ *E. coli*) and cytolethal distending toxin (CDT, from Campylobacter jejuni) induce DNA crosslinks or strand breaks. (2) Oxidative stress: Bacteria like *Staphylococcus aureus* and enterotoxigenic *Bacteroides* fragilis (ETBF) elevate intracellular reactive oxygen species (ROS) levels, exacerbating genomic instability. (3) Inhibition of DNA repair: Pathogens including *Helicobacter pylori* and high-risk human papillomavirus (HR-HPV) impair homologous recombination (HR) repair fidelity. (4) Immune activation: Microbial agonists (e.g., *Leptotrichia* DNA, *Faecalibacterium prausnitzii*) stimulate the cGAS-STING pathway or Toll-like receptors (TLRs), promoting dendritic cell (DC) maturation, CD8^+^ T-cell infiltration, and immunogenic cell death (ICD). Bottom panel (Radioresistance): Conversely, other microbial taxa promote tumor survival and therapy failure by: (1) Cell cycle progression: Activation of PI3K-AKT or Wnt/β-catenin signaling (e.g., by *Peptostreptococcus anaerobius* or *Salmonella* AvrA) drives G1/S transition, reducing the fraction of cells in the radiosensitive G2/M phase. (2) Immune evasion: Fungi like *Malassezia* recruit myeloid-derived suppressor cells (MDSCs), while butyrate-producing bacteria induce M2 macrophage polarization and regulatory T cells (Tregs), suppressing CD8^+^ T-cell effector functions. (3) Hypoxia exacerbation: Facultative anaerobes such as *Salmonella* Typhimurium suppress tumor angiogenesis, worsening hypoxia and diminishing the “oxygen fixation effect” of radiation. (4) Metabolic rewiring: Lactobacillus iners-derived L-lactate stabilizes HIF-1α and fuels glycolysis, while high fatty acid oxidation (FAO) activity supports DNA repair energetics. (5) Enhanced DNA repair: Upregulation of repair genes via microbial metabolites (e.g., butyrate) or ROS-induced mismatch repair (MMR) compromise. (6) Protective autophagy: Microbial lipopolysaccharide (LPS) triggers endoplasmic reticulum (ER) stress and HIF-1α/mitophagy axes, clearing damaged organelles and suppressing apoptosis. (7) Stemness maintenance: *Fusobacterium nucleatum* (Fn) activates Wnt/β-catenin signaling and metabolic adaptations that sustain cancer stem cell (CSC) populations, driving post-radiotherapy recurrence. The interplay between these opposing forces ultimately dictates whether radiation-induced DNA lesions are effectively converted into irreversible cell death or repaired, leading to therapy resistance. CSC, cancer stem cell; DC, dendritic cell; ER, endoplasmic reticulum; ETBF, enterotoxigenic *Bacteroides* fragilis; FAO, fatty acid oxidation; Fn, *Fusobacterium nucleatum*; HIF-1α, hypoxia-inducible factor 1 alpha; HR, homologous recombination; ICD, immunogenic cell death; LPS, lipopolysaccharide; MDSC, myeloid-derived suppressor cell; MMR, mismatch repair; ROS, reactive oxygen species; STING, stimulator of interferon genes; TLR, Toll-like receptor; Treg, regulatory T cell.

### 1.1 Distinction from gut microbiota

The intratumoral microbiota differs from the gut microbiota. The gut microbiota resides in the intestinal lumen, separated from the systemic circulation and most distal tumors by the intestinal epithelial barrier. Its metabolites-short-chain fatty acids, bile acids, and tryptophan catabolites-reach tumors through the circulation at low systemic concentrations, acting mainly via systemic immune priming and endocrine-like effects. In contrast, the intratumoral microbiota colonizes the tumor parenchyma, stroma, and even intracellular compartments directly. It produces metabolites locally within the tumor microenvironment, creating high local concentrations that enable direct crosstalk with tumor cells and infiltrating immune cells through metabolite sensing and pattern-recognition receptor signaling. While the gut microbiota influences the tumor microenvironment remotely via circulating factors and trafficking immune cells, intratumoral microbes are embedded within it and interact bidirectionally with cancer, stromal, endothelial, and immune cells *in situ*.

Although this review focuses on the tumoral microbiota, it is important to acknowledge that much of the current mechanistic understanding of microbiota-host interactions stems from gut microbiome studies. However, the extrapolation of these conclusions to the intratumoral environment should be cautious because of the essential differences: the lack of an epithelial barrier within the tumor, the distinct microbial burden and metabolic profile, the presence of direct cell-cell contact, and the fact that local concentrations of microbial metabolites are orders of magnitude higher than those that reach the tumor through the systemic circulation. Therefore, this review maintains a strict distinction between direct evidence derived from tumor-resident microbes and indirect clues inferred from the gut microbiome and emphasizes the urgency of conducting tumor-site-specific validation.

### 1.2 Experimental models and evidence classification

The studies discussed in this review encompass a range of experimental and clinical models, including human tumor specimens and clinical cohorts, cancer cell lines, patient-derived organoids, mouse and rat tumor models, and bioinformatic datasets. Because the experimental context influences the strength of evidence linking intratumoral microbiota to radiotherapy response, we considered both the model system and the presence or absence of radiotherapy when evaluating each study.

Accordingly, we classified the available evidence into three categories. Category (i) includes direct evidence from irradiated tumor models demonstrating altered radiosensitivity or radiotherapy response. Category (ii) includes mechanistic or *in vivo* evidence supporting a potential role in radiotherapy response but lacking direct validation in an irradiated tumor model. Category (iii) includes clinical associations, observational studies, or indirect mechanistic evidence without demonstrated causality. This framework was applied throughout the review to distinguish experimentally demonstrated effects from mechanistic inference and clinical correlation.

## 2 Effects on DNA damage and repair

### 2.1 Effect on DNA damage

#### 2.1.1 Intratumoral microbiota-produced toxins directly increase DNA damage

Toxins produced by the intratumoral microbiota can directly damage host cell DNA, thereby enhancing radiation-induced DNA injury and promoting tumor cell death. These toxins primarily induce DNA damage through two major mechanisms: DNA alkylation–mediated crosslinking and nuclease-mediated strand cleavage. Representative examples of these mechanisms are discussed below.

Certain *Escherichia coli* (e.g., *E. coli* NC101) strains carrying the *pks* island produce the genotoxin colibactin, which alkylates DNA to form interstrand crosslinks. These lesions may undergo spontaneous chemical degradation or be converted into double-strand breaks during DNA repair, thereby increasing the DNA damage burden in tumor cells (23, 24).

In addition, several Gram-negative pathogens, including *Campylobacter jejuni* (e.g., strain 81-176), *Salmonella enterica serotype Typhi* (e.g., strain Ty2), and *Haemophilus ducreyi* (e.g., strain 35000), produce cytolethal distending toxin (CDT). The active subunit CdtB exhibits DNase I-like nuclease activity, enabling it to induce DNA strand breaks in host cells. This genotoxic activity increases the cellular DNA damage burden and may enhance susceptibility to radiation-induced cytotoxicity (25–27).

In summary, intratumoral microbiota-derived toxins can directly induce DNA lesions or strand breaks, thereby amplifying radiation-induced DNA damage and enhancing therapeutic efficacy.

#### 2.1.2 Increased reactive oxygen species levels indirectly increase DNA damage

Ionizing radiation directly induces DNA strand breaks in tumor cells and simultaneously generates ROS through interactions with water molecules, thereby amplifying DNA damage and enhancing radiotherapy efficacy (28). In addition to radiation, intratumoral microorganisms can further increase DNA damage via ROS-mediated mechanisms. For instance, *Staphylococcus aureus* (e.g., strain USA300), a pathogen associated with squamous cell carcinoma and its precursor lesion, actinic keratosis, promotes excessive ROS and reactive nitrogen species production in tumor cells. These reactive molecules induce DNA double-strand breaks, as indicated by increased γ-H2AX phosphorylation. They also generate oxidative DNA lesions, including 8-hydroxy-2′-deoxyguanosine (8-OHdG), thereby contributing to genomic instability (29). Similarly, enterotoxigenic *Bacteroides fragilis* (e.g., strain BF9343) secretes *Bacteroides* fragilis toxin (BFT), which enhances ROS generation. Mechanistically, BFT upregulates spermine oxidase (SMO), a polyamine-catabolic enzyme that is highly responsive to inflammatory stimuli, leading to the production of large amounts of ROS. These ROS can activate the NLRP3 (NOD-like receptor family pyrin domain-containing protein 3) inflammasome and further induce DNA damage in colon epithelial cells (30–32).

Collectively, these findings suggest that the tumor microbiota can potentiate radiation-induced DNA damage either directly through microbial toxins or indirectly by increasing ROS levels, highlighting a complex interplay between the microbiome and radiotherapy response. In terms of evidence level, the genotoxic activities of colibactin and CDT and the ROS-mediated DNA-damaging effects of S. aureus and ETBF constitute causal mechanism evidence (category ii); however, direct experimental evidence that these tumor-resident microbes amplify radiation-induced cytotoxicity in irradiated tumor systems (category i) remains limited, because most of the supporting studies were conducted without irradiation.

### 2.2 Effect on DNA damage repair in tumor cells

The efficiency of the DNA damage response (DDR) is a major determinant of tumor cell sensitivity to radiotherapy. Tumor cells can survive radiation primarily through two strategies: defective checkpoint control, often due to TP53 mutations, which allows damage-tolerant replication, or enhanced repair proficiency, enabling the efficient resolution of radiation-induced lesions (33). Beyond these intrinsic mechanisms, emerging evidence indicates that the intratumoral microbiota can further modulate DDR through both direct and indirect pathways, thereby influencing overall radiosensitivity (22).

Gut-derived microbes can produce metabolites such as short-chain fatty acids, including butyrate, which may inhibit Histone deacetylase activity and alter the expression of DNA repair genes, promoting radioresistance (34, 35). Certain pathogens, including high-risk α-human papillomavirus (HR-HPV), *Chlamydia trachomatis* (e.g., serovar D strain D/UW-3/Cx), and *Helicobacter pylori*, can disrupt homologous recombination (HR) repair or shift repair toward error-prone nonhomologous end joining (NHEJ), thereby increasing genomic instability and modifying responses to radiation (36–38).

Additionally, microbial-induced ROS can impair DNA repair indirectly. For example, *Fusobacterium* species (e.g., *Fusobacterium nucleatum* ATCC 25586), enriched in colorectal cancer, activate ROS-generating pathways such as NADPH (nicotinamide adenine dinucleotide phosphate) oxidase and TLR4–NF-κB (Toll-like receptor 4, nuclear factor kappa-light-chain-enhancer of activated B cells) signaling. ROS can accumulate as oxidative DNA lesions (e.g., 8-oxoguanine) in regions that are difficult to repair, compromise mismatch repair (MMR), and lead to persistent DNA damage (39–42).

In summary, the tumor microbiota can influence DDR through both direct modulation of repair pathways and indirect ROS-mediated mechanisms, affecting the overall radiosensitivity of tumor cells. Within our evidence framework, the modulation of DDR by microbial metabolites and the impairment of HR by HR-HPV, Chlamydia trachomatis, or H. pylori are supported by causal mechanism evidence (category ii) established largely in non-irradiated or extra-tumoral systems, whereas their translation to intratumoral radiation response currently represents indirect or extrapolated evidence (category iii) that requires tumor-site-specific validation.

Despite the accumulating evidence linking intratumoral microbes to DDR modulation, several critical caveats warrant consideration. First, the net effect of microbial exposure on DDR appears to be highly context-dependent. For instance, while *H. pylori* infection impairs HR repair in gastric epithelial cells (38), other studies have suggested that chronic *H. pylori* infection may paradoxically upregulate certain repair genes as a host compensatory response, complicating the interpretation of its overall impact on radiosensitivity. Similarly, although butyrate-producing bacteria are generally considered to promote radioresistance via HDAC inhibition (34, 35), the concentration-dependent and cell-type-specific effects of butyrate on DDR gene expression remain poorly defined, with some reports indicating that low-dose butyrate may actually sensitize rather than protect tumor cells.

The methods employed to study microbe–DDR interactions present inherent limitations. Most mechanistic insights derive from *in vitro* co-culture systems using single bacterial strains, which fail to recapitulate the polymicrobial complexity of the actual tumor microenvironment. Moreover, distinguishing the direct effects of intratumoral microbes on DDR from indirect effects mediated by host inflammatory responses or metabolic alterations remains methodologically challenging. Many studies rely on pharmacological inhibitors or genetic knockouts that may have off-target effects, and the use of antibiotic-treated animal models can inadvertently alter gut microbiota composition, introducing confounding variables that complicate causal attribution (22).

The translational relevance of these findings to clinical radiotherapy remains uncertain. Most existing evidence comes from preclinical models, and the extent to which microbial modulation of DDR translates into meaningful differences in patient outcomes has yet to be systematically evaluated. Given these limitations, future studies should prioritize longitudinal sampling in patients receiving radiotherapy to establish temporal relationships between microbial dynamics and DDR activity, as well as the use of gnotobiotic models to isolate the contributions of specific microbial taxa.

### 2.3 Integrated perspective on net radiosensitivity

Importantly, the net radiosensitivity outcome depends not on the mere presence of microbial-induced DNA damage or repair modulation, but on the temporal integration of these two opposing forces. This integration is strongly influenced by temporal differences between DNA damage and repair modulation. Radiation and microbial genotoxins can trigger an acute burst of DNA lesions within minutes. In contrast, changes in DNA repair activity typically emerge over several hours. If critical repair factors, such as HR or nucleotide excision repair (NER) complexes, are concurrently inhibited during this early window, persistent lesions accumulate and favor cell death; conversely, if microbial metabolites such as butyrate pre-upregulate repair reserves prior to irradiation, tumor cells may acquire relative radioresistance. Beyond temporal dynamics, substrate-specific matching between the type of DNA lesion and the affected repair pathway critically determines the net outcome. Colibactin primarily generates interstrand crosslinks that rely on NER and HR, whereas ROS predominantly induce base oxidation and single-strand breaks that are resolved by base excision repair (BER). The strongest radiosensitizing synergy may occur when a microbe both generates a specific type of DNA lesion and suppresses the corresponding repair pathway. However, this interaction remains poorly characterized in current experimental systems. Spatial heterogeneity of microbial colonization within the tumor adds another layer of complexity, as distinct niches, such as hypoxic cores versus invasive margins, may harbor functionally opposing microbial communities; we hypothesize that the overall tumor response dependent on the ecologically dominant niche rather than on the averaged effects of all taxa.

Collectively, these considerations underscore that future mechanistic investigations should employ spatially resolved multi-omics approaches and real-time damage-tracking models to decipher how these interdependent factors collectively shape individualized radiotherapy outcomes.

### 2.4 Effect on the tumor cell cycle

Cell cycle regulation is closely associated with radiosensitivity. Alterations in cell cycle progression can directly influence the response of tumor cells to radiotherapy, as different phases exhibit distinct capacities for DNA damage recognition and repair.

During the S phase, cells actively replicate their DNA and rely predominantly on HR, a high-fidelity repair mechanism that enables precise correction of DNA lesions. Consequently, cells in S phase generally exhibit increased resistance to radiation. In contrast, cells in the G1 phase possess relatively limited repair capacity. Mild DNA damage can be resolved through checkpoint activation mediated by proteins such as p53, whereas severe damage often triggers apoptosis, resulting in moderate radiosensitivity. Cells in the G2/M phase are the most radiosensitive, as DNA repair capacity is reduced, particularly during mitosis, when repair processes are largely suppressed (43–45).

Emerging evidence indicates that intratumoral microorganisms can modulate tumor cell cycle progression through the regulation of key signaling pathways. For example, *Peptostreptococcus anaerobius* (e.g., strain ATCC 27337) binds to integrins α_2_/β_1_, which are highly expressed on colorectal cancer cells, via its surface protein putative cell wall binding repeat 2. This interaction activates focal adhesion kinase phosphorylation and subsequently stimulates both the PI3K–Akt(phosphatidylinositol 3-kinase,protein kinase B) and NF-κB signaling pathways (46). Activation of the PI3K–Akt pathway inhibits glycogen synthase kinase-3β through phosphorylation, thereby stabilizing cyclin D1 and promoting the G1-to-S phase transition (47). In parallel, NF-κB signaling upregulates the expression of key cell cycle regulators, including cyclin D1, further facilitating cell cycle progression (48, 49). In addition, *Chlamydia trachomatis* infection has been shown to disrupt protein phosphatase 2A (PP2A) activity and activate checkpoint kinase 2 (Chk2), leading to cell cycle arrest at the G2/M phase. Given that tumor cells exhibit maximal radiosensitivity during the G2 and M phases, such infection-induced G2/M arrest may enhance the sensitivity of tumor cells to radiotherapy (37).

The *Salmonella* (e.g., strain SL1344) effector protein AvrA has also been implicated in the regulation of cell cycle progression through activation of the Wnt/β-catenin signaling pathway. AvrA possesses deubiquitinase activity and stabilizes β-catenin by removing ubiquitin moieties. This promotes β-catenin accumulation and facilitates its phosphorylation and nuclear translocation. Concurrently, AvrA upregulates upstream components of the Wnt pathway as well as transcription factors such as TCF/LEF1. These events lead to activation of β-catenin-dependent transcriptional programs and increased expression of canonical target genes, including c-Myc (MYC proto-oncogene) and cyclin D1, ultimately promoting the G1-to-S phase transition and enhancing cell proliferation. Furthermore, given the critical role of the Wnt/β-catenin pathways in DNA repair, cell cycle regulation, and radiotherapy response, AvrA-mediated activation of these pathways is hypothesized to reduce tumor radiosensitivity. However, direct experimental evidence supporting this mechanism remains limited and warrants further investigation (50, 51).

In summary, intratumoral microorganisms can regulate key signaling pathways involved in cell cycle control. Through these mechanisms, they may directly alter tumor cell radiosensitivity or indirectly influence DNA damage repair capacity, ultimately modulating the therapeutic efficacy of radiotherapy. Regarding evidence levels, the cell-cycle effects of P. anaerobius and C. trachomatis rest on causal mechanism evidence (category ii), whereas the proposed AvrA-mediated radioresistance is currently supported only by indirect or extrapolated evidence (category iii); direct experimental confirmation in irradiated tumor models is required.

## 3 Intratumoral microbes influence the tumor immune microenvironment

The tumor immune microenvironment (TIME) refers to the functional ecosystem within and surrounding tumor lesions and is composed of tumor cells, various immune cell subsets, nonimmune stromal cells, cytokines/chemokines, metabolic products, and physical factors such as hypoxia, acidity, and interstitial pressure. Through the coordinated actions of diverse immune cells and cytokine networks, the TIME critically modulates the therapeutic efficacy of radiotherapy (52–54). CD8^+^ T cells serve as the key effector population responsible for eliminating residual tumor cells, and both their intratumoral infiltration density and activation status in peripheral blood are positively correlated with the therapeutic efficacy of radiotherapy (55, 56). Macrophage polarization exerts bidirectional regulatory effects: M1 macrophages increase the efficacy of radiotherapy through proinflammatory activity and the activation of CD8^+^ T cells, whereas M2 macrophages attenuate therapeutic responses by releasing anti-inflammatory mediators and proangiogenic factors (57). As a central bridge between innate and adaptive immunity, dendritic cells (DCs) rely on their antigen-presenting efficiency to ensure that radiotherapy-induced immunogenic cell death effectively activates CD8^+^ T-cell responses (58). Radiotherapy-induced immunogenic cell death (ICD) results in the release of tumor antigens and Damage-associated molecular patterns (DAMPs), which are cross-presented by dendritic cells to activate CD8^+^ T cells; when DC function is impaired, the adaptive antitumor immunity elicited by radiotherapy is substantially compromised (59). Moreover, the cytokine profile serves as an important regulatory layer—proinflammatory cytokines such as TNF-α (tumor necrosis factor-alpha) and IL-12 (interleukin-12) increase radiosensitivity and promote immune cell infiltration (60, 61), whereas anti-inflammatory cytokines such as TGF-β (transforming growth factor-beta) and IL-10 (interleukin-10) suppress T-cell and dendritic cell functions, hinder the initiation of antitumor immunity, and ultimately weaken the therapeutic efficacy of radiotherapy (54, 62, 63). Taken together, the effector functions of CD8^+^ T cells, along with their interplay with macrophage polarization, DC-mediated antigen presentation, and the surrounding cytokine network, constitute the central regulatory framework underlying radiotherapy-induced immune responses. Being the TIME features dependent on the immune cell composition, activation states, and cytokine profiles (63, 64), the regulatory effects of intratumoral microorganisms in reshaping the TIME may modulate tumor radiosensitivity (65).

The regulatory effects of intratumoral microbes on the TIME are primarily orchestrated through three core innate immune sensing pathways—the STING (stimulator of interferon genes), NLRP3 (NOD-, LRR-, and pyrin domain-containing protein 3) inflammasome, and TLR4 (Toll-like receptor 4) pathways—which collectively determine the outcome of radiotherapy-induced ICD. Depending on the microbial taxa and the specific molecular patterns they present, these pathways can be either activated to promote antitumor immunity or hijacked to establish an immunosuppressive milieu that dampens radiotherapy efficacy.

### 3.1 Enhancing the immune response via activation of innate immune pathways

Intratumoral microbes that activate STING, NLRP3, or TLR4 signaling can potentiate the immunogenic effects of radiotherapy by promoting DC maturation, enhancing antigen cross-presentation, and facilitating CD8^+^ T-cell infiltration.

The cGAS–STING pathway serves as a sentinel for cytosolic microbial DNA and host-derived double-stranded DNA released during radiotherapy-induced ICD. Upon activation, STING triggers the production of type I interferons (IFN-I), which are essential for DC maturation and cross-priming of CD8^+^ T cells. *Faecalibacterium prausnitzii* (e.g., strain A2-165) has been reported to modulate DC function; however, a specific STING–IFN-I axis that increases DC antigen-presenting efficiency and CTL infiltration in the intratumoral setting is not yet established, and tolerogenic (IL-10/IDO) DC effects of F. prausnitzii have also been reported (65). Similarly, microbial DNA from Leptotrichia species (e.g., *Leptotrichia buccalis*) may engage the cGAS–STING pathway and could synergize with radiation-induced DNA damage to amplify IFN-I signaling; direct experimental evidence for this mechanism is currently lacking, but the enrichment of *Leptotrichia* in early-stage head and neck tumors and its association with favorable clinical outcomes provide indirect support for an immunostimulatory role (66).Notably, the STING pathway also mediates the abscopal effect of radiotherapy, and its microbial activation may extend therapeutic benefits beyond the irradiated field. However, the precise molecular mechanisms by which specific intratumoral bacterial DNA sequences engage STING in the context of radiotherapy remain an active area of investigation.

The NLRP3 inflammasome constitutes another critical checkpoint in the radiotherapy–immune axis. Microbial components, including bacterial DNA, peptidoglycan, and cell wall fragments, can activate the NLRP3 inflammasome via intracellular signaling, leading to caspase-1 activation and the maturation and release of IL-1β and IL-18 (65).*Fusobacterium nucleatum* (e.g., ATCC 25586) and other Gram-negative anaerobes can provide the “second signal” required for NLRP3 assembly through their LPS (lipopolysaccharide) and other microbe-associated molecular patterns (MAMPs) (67, 68).IL-18 produced downstream of NLRP3 activation may enhance DC sensing of CRT–HMGB1–ATP signals released by dying tumor cells. IL-1β may further promote an inflammatory niche that facilitates CD8 T-cell infiltration through the CXCL9/10–CXCR3 axis. Together, these effects may contribute to an “immune-hot” tumor microenvironment (67). Importantly, radiotherapy-induced ATP release serves as a canonical NLRP3 agonist (67); when combined with microbial priming, this dual activation creates a feed-forward loop that amplifies ICD-driven antitumor immunity.

The TLR4 pathway is engaged by microbial LPS and other lipid-based MAMPs, leading to MyD88/NF-κB-dependent proinflammatory cytokine production. F. nucleatum can bind to TLR4 through its LPS, activating the MyD88/NF-κB pathway and promoting the release of proinflammatory factors such as IL-1β and TNF-α (65).In head and neck squamous cell carcinoma, *Leptotrichia* (e.g., *L. buccalis*) was significantly enriched in early-stage (T1–T2) tumors compared with advanced-stage (T3–T4) tumors and was associated with prolonged overall survival, suggesting a protective role within the tumor microenvironment (66).Whether this favorable association reflects an immune-active, ‘immune-hot’ TIME—and whether such immune activation could enhance ICD-induced antitumor immunity and thereby increase radiotherapy sensitivity—remains to be directly demonstrated.

Collectively, these three pathways—STING, NLRP3, and TLR4—are not mutually exclusive but converge on the enhancement of DC-mediated antigen presentation and the establishment of a T-cell-inflamed tumor microenvironment. Importantly, the efficacy of their activation depends on the timing and context of microbial exposure relative to radiation delivery. In preclinical models, the combination of radiotherapy with microbial STING agonists or TLR4 ligands has been shown to produce synergistic antitumor effects; however, clinical translation requires careful consideration of the optimal microbial ligands and their intratumoral delivery. In terms of evidence classification, the activation of STING, NLRP3, and TLR4 pathways by intratumoral bacteria to promote antitumor immunity currently constitutes indirect or extrapolated evidence (category iii), because a direct cGAS–STING–IFN-I link for intratumoral bacteria is not yet established; although the NLRP3 and TLR4 axes are supported by causal mechanism evidence for specific microbes (e.g., F. nucleatum LPS) (category ii), their combination with radiotherapy has produced synergistic antitumor effects in some preclinical models (category i), whereas the human correlative data (e.g., intratumoral Leptotrichia in head and neck cancer) remain category (iii) evidence.

Bacteriolytic therapy with anaerobic bacteria also exemplifies the potentiation of radiotherapy through immune activation. Bettegowda et al. demonstrated that combination therapy with Clostridium novyi-NT and radiotherapy produced synergistic antitumor effects by exploiting the hypoxic microenvironment of tumors. C. novyi-NT selectively germinates within hypoxic tumor regions, which are relatively resistant to radiotherapy, thereby complementing radiation-induced tumor cell killing and enhancing overall antitumor efficacy (69). Agrawal et al. revealed that infection with *Clostridium novyi-NT* within hypoxic tumor regions induces the recruitment of innate immune cells such as neutrophils and macrophages, accompanied by the release of proinflammatory cytokines, including IL-6. Moreover, *C. novyi-NT* infection triggers the activation of adaptive immunity by promoting CD8^+^ T-cell infiltration into the tumor. This immunostimulatory effect can reverse the immunosuppressive state of the hypoxic tumor microenvironment following radiotherapy, synergistically amplifying the ICD-induced antitumor memory response and markedly reducing tumor recurrence (70).

### 3.2 Suppression of the immune response via innate immune pathway subversion and immunosuppressive cell recruitment

Conversely, certain intratumoral microbes can subvert STING, NLRP3, and TLR4 signaling to promote an immunosuppressive TIME, thereby impairing the immunogenic effects of radiotherapy. These inhibitory mechanisms operate through three interrelated layers: (1) disruption of DC antigen-presenting function; (2) recruitment and functional polarization of immunosuppressive cells (MDSCs, Tregs, and M2 macrophages); and (3) attenuation of CD8^+^ T-cell effector functions.

Subversion of TLR signaling represents one such mechanism. Whereas TLR4 activation by F. nucleatum LPS can be immunostimulatory under some conditions, chronic or aberrant TLR4 signaling driven by other microbial taxa can lead to immunosuppressive outcomes. For example, *Malassezia* (e.g., *Malassezia globosa*), an important pathogenic fungus implicated in pancreatic cancer, activates the lectin (MBL) complement cascade, with downstream complement-fragment release; this pathway recruits MDSCs and can suppress antigen-presenting-cell activation, thereby inhibiting the initiation of adaptive immune responses (71).

Interference with the STING–IFN-I axis can also be co-opted by microbes to evade immune detection. Some intratumoral bacteria have been reported to degrade cytosolic DNA via secreted nucleases, thereby reducing the availability of STING agonists and dampening IFN-I production. This mechanism, if operative in the tumor microenvironment, could blunt the STING-dependent DC activation that is critical for the cross-priming of CD8^+^ T cells following radiotherapy. Although direct evidence linking specific intratumoral microbes to STING pathway inhibition in irradiated tumors is still emerging, it represents a potentially important mechanism of radioresistance that warrants further investigation.

NLRP3 inflammasome dysfunction induced by certain microbes can also suppress antitumor immunity. Rather than activating the inflammasome, certain microbial metabolites, including butyrate and other short-chain fatty acids, can suppress NLRP3 signaling (e.g., via GPR109A- and GPR43-dependent mechanisms), leading to reduced IL-1β and IL-18 production (14).Furthermore, butyrate promotes an anti-inflammatory macrophage phenotype and regulatory T-cell differentiation in gut-microbiota studies (14); whether intratumoral butyrate-producing bacteria (e.g., *Clostridium butyricum*) likewise enhance M2 macrophage polarization, inhibit CD8^+^ T-cell infiltration, or compromise anti-PD-1 therapy in the tumor microenvironment warrants direct investigation. The impaired NLRP3-dependent cytokine milieu diminishes the capacity of DCs to sense and respond to ICD-associated DAMPs, effectively interrupting the immunogenic signaling cascade.

Recruitment of immunosuppressive cells is a hallmark of microbe-driven immune evasion. *Malassezia*-mediated complement activation leads to the recruitment of MDSCs into pancreatic tumor tissues. These MDSCs exert potent immunosuppressive effects by depleting essential amino acids required for immune cell function (such as arginine), producing nitric oxide and ROS, and upregulating PD-L1 expression. Collectively, these mechanisms suppress the proliferation and cytotoxic activity of effector T cells (including CD4^+^ Th1 cells and CD8^+^ cytotoxic T lymphocytes), thereby establishing an immunosuppressive loop that facilitates PDAC progression (71, 72). In addition, certain commensal *Clostridium* species (e.g., *Clostridium butyricum*) can metabolically produce short-chain fatty acids, which activate GPR43 signaling and promote regulatory T-cell (Treg) differentiation (14, 73). Given that Tregs are a major source of TGF-β, this pathway may contribute to the TGF-β–enriched immunosuppressive tumor microenvironment that dampens radiotherapy-induced antitumor immunity. TGF-β not only suppresses the expression of perforin and granzyme B in CD8^+^ T cells but also induces the upregulation of indoleamine 2,3-dioxygenase(IDO), leading to tryptophan depletion and kynurenine accumulation, which together contribute to T-cell functional exhaustion (74, 75).

The overall consequence of these immunosuppressive mechanisms is that even when radiotherapy successfully induces calreticulin exposure and HMGB1 release, DCs fail to accomplish effective antigen cross-presentation because of the immunosuppressive TGF-β/IDO-enriched microenvironment. Consequently, the signaling cascade of ICD is effectively “interrupted,” rendering the tumor in a state of “pseudo-hot”—characterized by the presence of immune cells that are functionally exhausted and incapable of mounting an effective antitumor response (76, 77).This disruption of the DC–CD8^+^ T-cell axis is a critical determinant of radiotherapy resistance and highlights the need for combinatorial strategies that reprogram the TIME toward a state conducive to ICD-driven antitumor immunity.

In summary, the net effect of intratumoral microbes on the TIME is determined by the balance between STING/NLRP3/TLR4 pathway activation (driving DC maturation, CD8^+^ T-cell infiltration, and memory formation) and pathway subversion (leading to DC dysfunction, MDSC/Treg recruitment, and CD8^+^ T-cell exhaustion). This complex interplay underscores the importance of understanding the precise microbial composition and functional state within the tumor microenvironment when designing strategies to enhance radiotherapy efficacy. The immunosuppressive mechanisms—including Malassezia-mediated complement activation and NLRP3 subversion—are supported by causal mechanism evidence (category ii) derived largely from non-irradiated models, whereas butyrate-driven GPR43–TGF-β signaling derives from gut-microbiota studies and constitutes indirect or extrapolated evidence (category iii); all of these mechanisms are extrapolated to the radiotherapy setting (category iii).

The net effect of intratumoral microbes on the TIME is determined by the balance between STING/NLRP3/TLR4 pathway activation and subversion. However, this binary classification—”immunostimulatory” versus “immunosuppressive” microbes—oversimplifies a highly dynamic and context-dependent process. Several layers of complexity warrant critical consideration.

The temporal dynamics of microbial–immune interactions are poorly understood. A microbe that primes the STING pathway and promotes CD8^+^ T-cell infiltration during the early phase of infection may subsequently recruit immunosuppressive MDSCs as a result of chronic inflammation, shifting the TIME toward an exhausted phenotype. Thus, the outcome likely depends on the duration and timing of microbial exposure relative to radiation delivery, an aspect rarely captured in static preclinical models.

The spatial heterogeneity of microbial colonization within tumors introduces additional uncertainty. While certain bacterial taxa may cluster in hypoxic, necrotic regions where they activate local innate immunity, adjacent tumor zones may harbor different microbial communities with opposing effects on DC maturation or T-cell function. Conventional bulk sequencing averages these regional differences, obscuring the spatial architecture that ultimately dictates the integrated immune response to radiotherapy.

The functional redundancy and crosstalk among the STING, NLRP3, and TLR4 pathways mean that activating one pathway may compensate for the inhibition of another. For instance, TLR4-driven NF-κB signaling can synergize with STING–IFN-I to enhance DC cross-presentation, but excessive NF-κB activation may also upregulate PD-L1 on tumor cells and myeloid cells, dampening the eventual CD8^+^ T-cell effector phase. This pleiotropy complicates the interpretation of pathway-specific interventions and suggests that targeting a single microbial ligand may produce unpredictable results.

Consequently, the current literature may underestimate the net immunosuppressive effect of certain microbes by focusing on proximal signaling events while neglecting downstream homeostatic counter-regulations. A more integrated approach—combining metagenomic profiling, spatial transcriptomics, and dynamic immune monitoring—is urgently needed to clarify whether a given microbial signature truly predicts radiosensitization or radioresistance in individual patients.

## 4 Regulation of tumor cell metabolism

Intratumoral microorganisms can reprogram the fundamental metabolic pathways of tumor cells, including glucose and lipid metabolism, thereby modulating their responsiveness and sensitivity to radiotherapy. This metabolic reprogramming primarily occurs through the modulation of the energy supply, macromolecule biosynthesis, and redox homeostasis, which are tightly associated with both radioresistance and radiosensitivity (78).

### 4.1 Glucose metabolic reprogramming

In terms of glucose metabolism, intratumoral microbes can influence the response to radiotherapy by modulating aerobic glycolysis, also known as the Warburg effect. In a multiomics analysis and *in vitro* study involving 101 patients with locally advanced cervical cancer, Lauren E. Colbert and colleagues identified that the intratumoral enrichment of *Lactobacillus iners* (e.g., strain ATCC 55195) was an independent predictor of a poor response to chemoradiotherapy. The experimental evidence further demonstrated that exogenous supplementation with L-lactate, rather than D-lactate, sodium lactate, or other lactate isomers, was sufficient to induce radioresistance in cervical cancer cell lines (HeLa, SiHa, and CaSki) and patient-derived organoids (PDOs), establishing L-lactate as the key metabolic mediator of treatment resistance. *Lactobacillus iners* modulates intracellular lactate signaling and the expression of related metabolic genes in tumor cells through its secretion of L-lactate and other metabolites. L-Lactate can regulate and stabilize hypoxia-inducible factor 1α (HIF-1α), which in turn upregulates the expression of glycolysis-related genes. This HIF-1α-driven transcriptional program enables tumor cells to sustain high glycolytic flux even under hypoxic conditions, thereby generating ATP to support cellular survival and proliferation. Tumors enriched with Lactobacillus iners exhibit significantly higher intracellular ATP levels than L. iners-negative tumors. Cervical cancer cells pretreated with L-lactate (e.g., primary B1188 and HeLa cells) generate 1.5- to 2-fold more ATP under hypoxic conditions than control cells, indicating that enhanced glycolytic activity effectively compensates for hypoxia-induced energy deficiency. On the one hand, sufficient ATP supplies energy for DNA repair enzymes such as PARP [poly (ADP-ribose) polymerase] and DNA polymerases, facilitating the repair of chemoradiotherapy-induced DNA double-strand breaks. On the other hand, pyruvate generated from glycolysis participates in the “pyruvate–lactate cycle” to maintain intracellular pH homeostasis, thereby preventing excessive acidification that would otherwise exacerbate treatment-induced apoptosis. *In vitro*, cervical cancer cells pretreated with L-lactate exhibited markedly faster clearance of the DNA damage marker γ-H2AX and a 30–50% increase in cell viability under combined hypoxic and irradiation conditions, providing direct evidence that increased glycolysis facilitates the repair of radiation-induced damage in hypoxic environments (79).

In addition, infection with Kaposi’s sarcoma-associated herpesvirus (KSHV, e.g., strain GK18) can also induce sustained glycolysis by upregulating HIF-1α expression and activating the PI3K/AKT/mTOR signaling pathway, thereby contributing to radioresistance (80). Moreover, intratumoral facultative anaerobes can upregulate the expression of glucose transporters in tumor cells, thereby increasing glucose uptake and accelerating lactate production. The resulting acidic microenvironment not only reduces the efficiency of radiation-induced DNA damage but also impairs effector T-cell function, ultimately weakening antitumor immune clearance (81).

*Human cytomegalovirus* (HCMV, e.g., strain AD169) markedly upregulates the activity of glycolytic pathways, the tricarboxylic acid cycle, and fatty acid biosynthesis. This virus may influence the tumor energy supply and microenvironmental acidification by modulating glycolysis and lactate metabolism, which could theoretically affect radiotherapy sensitivity; however, direct evidence remains to be established (82, 83). In terms of evidence levels, the L. iners–L-lactate axis represents direct experimental evidence (category i), because radioresistance was demonstrated in irradiated cervical cancer cell lines and patient-derived organoids, whereas the proposed contributions of KSHV and HCMV to radioresistance remain indirect or extrapolated evidence (category iii), as direct evidence has yet to be established.

### 4.2 Lipid metabolic reprogramming

By analyzing intratumoral microbiome and host transcriptome data from 420 patients with colon adenocarcinoma in the TCGA (The Cancer Genome Atlas) database, Liu et al. found that patients stratified into high and low fatty acid metabolism (FAM) groups based on Gene Set Variation Analysis scores exhibited significant differences in intratumoral microbial community composition. At the genus level, the two subtypes were clearly separated in t-SNE(t-Distributed Stochastic Neighbor Embedding) space, with the difference confirmed to be statistically significant by PERMANOVA(Permutational Multivariate Analysis of Variance). The high FAM group was enriched in genera such as *Desulfovibrio* (e.g., *D. vulgaris*), *Desulfotomaculum* (e.g., *D. nigrificans*), *Streptococcus* (e.g., *S. gallolyticus*), *Mycobacterium* (e.g., *M. avium*), and *Desulfobacterium* (e.g., *D. autotrophicum*). These findings provide evidence supporting an association between the intratumoral microbiome and lipid metabolism in COAD (colon adenocarcinoma). Moreover, the high FAM group exhibited significantly higher fatty acid oxidation (FAO) activity, a key component of lipid metabolism, suggesting that tumor cells in this subtype may potentially acquire additional energy to cope with environmental stress through enhanced FAO (84). FAO functions as a critical regulatory pathway in the repair of DNA double-strand breaks, particularly through HR, thereby facilitating DNA damage repair and preserving genomic stability (85). The increased DNA damage repair capacity observed in the high FAM expression group may consequently attenuate the therapeutic efficacy of radiotherapy.

Furthermore, the high FAM expression group exhibited significantly higher Tumor Immune Dysfunction and Exclusion (TIDE) scores, indicating stronger immune evasion potential, along with reduced immune cell infiltration, including decreased abundances of CD4^+^ and CD8^+^ T cells. This immune phenotype is closely associated with microbe-regulated lipid metabolism—intratumoral microorganisms may increase fatty acid oxidation, thereby depleting the amount of lipid substrates available to immune cells within the tumor microenvironment (TME) and leading to immune cell dysfunction and exhaustion (86). The therapeutic efficacy of radiotherapy depends on both the direct cytotoxic effects on tumor cells and the clearance of tumor debris, as well as the induction of antitumor immune responses by immune cells within the tumor microenvironment (87). If intratumoral microbes suppress immune cell infiltration through lipid metabolic reprogramming, they may weaken postradiotherapy antitumor immunity and ultimately compromise overall treatment efficacy. Within the evidence framework, the association between intratumoral microbial composition and fatty acid metabolism currently rests on correlative bioinformatic analysis of TCGA data (category iii), whereas the FAO–PARP1–DSB repair axis represents causal mechanism evidence (category ii) established in non-microbial systems.

## 5 Tumor microenvironment remodeling aggravates hypoxia

Tumor hypoxia markedly *reduces* the efficacy of radiotherapy through multiple mechanisms, which can be broadly categorized into direct and indirect effects. Directly, hypoxia weakens the “oxygen fixation effect,” thereby reducing the efficiency of radiation-induced DNA damage. It also reprograms DNA damage response pathways by activating ATM/ATR (ataxia telangiectasia mutated/ATM-Rad3-related) signaling while suppressing RAD51 (DNA repair protein RAD51) and BRCA1/2 (breast cancer susceptibility protein 1/2) to induce cell cycle arrest and facilitate the formation of radioresistant clones, ultimately decreasing radiosensitivity. Indirectly, the hypoxic microenvironment increases the number of radioresistant cancer stem cells (CSCs) and remodels the immune landscape, promoting the recruitment of immunosuppressive cells such as M2 macrophages. Concurrently, HIF-mediated metabolic and angiogenic programs activate glycolysis, angiogenesis, and extracellular matrix remodeling, sustaining tumor survival and invasion under low-oxygen conditions. Clinical evidence further demonstrates that patients with hypoxic tumor regions exhibit significantly higher rates of local recurrence and mortality, as well as reduced long-term survival following radiotherapy, highlighting hypoxia as a critical biological barrier limiting radiotherapeutic efficacy (88–90).

*Salmonella enterica serovar Typhimurium* (a member of the same genus as S. Typhi) can sense tumor-derived metabolites, such as aspartate and ribose released from necrotic tissue, and actively migrate toward hypoxic tumor regions via flagella-mediated motility. It preferentially colonizes tumors at levels more than 1,000-fold higher than those in normal tissues (91). Studies have demonstrated that S. Typhimurium suppresses tumor angiogenesis and exacerbates tumor hypoxia through dual mechanisms. S. Typhimurium has been reported to suppress the AKT/HIF-1α/VEGF axis and inhibit tumor angiogenesis (92). Consistently, an attenuated S. typhimurium VNP20009-based system was shown to downregulate VEGF-A and VEGFR-2, accompanied by reduced angiogenic activity in melanoma models (93).In parallel, *S. Typhimurium* disrupts endothelial cell function, either through the secretion of bioactive factors or via modulation of the tumor microenvironment. For instance, it inhibits the proliferation of human microvascular endothelial cells (HMEC-1), leading to a marked decrease in tumor microvessel density. Collectively, these effects impair oxygen and nutrient delivery, thereby aggravating intratumoral hypoxia and promoting radioresistance (91, 92). These findings highlight a previously underappreciated role of tumor-colonizing bacteria in reshaping the hypoxic tumor microenvironment and modulating radiotherapeutic response.

In contrast, *Clostridium novyi-NT*, an attenuated obligate anaerobe, represents a unique hypoxia-targeting strategy with a predominantly radiosensitizing effect. Its spores selectively germinate in hypoxic tumor regions and directly lyse radioresistant hypoxic tumor cells, thereby enhancing the cytotoxic effects of radiotherapy (69). In addition, C. novyi-NT infection recruits innate and adaptive immune cells—including neutrophils, macrophages, and CD8^+^ T lymphocytes—further amplifying antitumor immune responses and synergizing with radiation-induced immunogenic cell death (70). Preclinical studies have demonstrated that combining *C. novyi-NT* with radiotherapy leads to significant tumor regression and improved survival across multiple tumor models, highlighting its potential as an effective radiosensitizing agent. Regarding evidence levels, the angiogenesis-suppressing and hypoxia-aggravating effects of S. Typhimurium constitute *in vivo* causal mechanism evidence (category ii), whereas their translation into radioresistance rests in part on extrapolation from the hypoxia–radioresistance literature (category iii); by contrast, the combination of C. novyi-NT with radiotherapy provides direct experimental evidence of radiosensitization (category i).

## 6 Regulation of tumor cell autophagy

Radiotherapy-induced DNA double-strand breaks, together with the subsequent increase in ROS production, rapidly trigger the DNA damage response in tumor cells. During this process, the ATM/ATR–CHK2–BECN1(beclin 1) axis functions as a “gatekeeper.” Activated ATM not only phosphorylates CHK2 to arrest cells at the G2/M checkpoint but also phosphorylates Beclin-1, thereby recruiting the class III PI3K complex and rapidly initiating autophagosome formation (94). This form of “protective autophagy” can eliminate damaged mitochondria, micronuclei, and excessive ROS within a few hours, thereby maintaining ATP and NADPH homeostasis and ultimately attenuating the cytotoxic effects of radiotherapy (95). However, autophagy is not merely a “prosurvival” mechanism; *its dual role confers* a complex function in determining tumor radiosensitivity. Under stress conditions such as nutrient deprivation or glutamine exhaustion, moderate autophagy mediated by ATG5 (autophagy-related protein 5) or ATG7 (autophagy-related protein 7) can amplify apoptotic signaling, thereby increasing the sensitivity of tumor cells to radiotherapy (96). In contrast, under conditions of p53 deficiency or sustained mTOR activation, autophagy can be hijacked as an “escape” pathway through which pro-apoptotic proteins are degraded and the mitochondrial membrane potential is stabilized, ultimately leading to the development of radioresistance (97).

Emerging evidence indicates that intratumoral facultative anaerobes, such as members of the Enterobacteriaceae family (e.g., *Escherichia coli*), can exacerbate local hypoxia primarily through metabolic oxygen consumption. The resulting oxygen depletion inhibits prolyl hydroxylase-mediated hydroxylation of HIF-1α, thereby promoting HIF-1α stabilization. Stabilized HIF-1α then transcriptionally activates BNIP3 (BCL2 interacting protein 3) and NIX(NIP3-like protein X), inducing mitophagy. This mitophagy reduces intracellular ROS levels, limits radiotherapy-induced oxidative damage and apoptosis, and ultimately confers radioresistance (89, 98, 99).

In non-small cell lung cancer tissues (NSCLC), multiple gene clusters of the gram-negative bacterium *Escherichia–Shigella* (e.g., *Escherichia coli* or *Shigella flexneri*) encode LPS synthases, and the intratumoral production of LPS is markedly higher in these tissues than in adjacent nontumor tissues (100). LPS can trigger endoplasmic reticulum (ER) stress in tumor cells, activating two branches of the unfolded protein response, the IRE1α–JNK–Beclin1(Inositol-requiring enzyme 1α,c-Jun N-terminal kinase, Beclin 1) and PERK–eIF2α–ATF4(Protein kinase R-like endoplasmic reticulum kinase, Eukaryotic initiation factor 2α, Activating transcription factor 4) pathways, which upregulate the expression of autophagy-related proteins. Persistent ER stress and the subsequent autophagic cycle facilitate the removal of damaged organelles and suppress apoptosis, ultimately contributing to the development of radioresistance (101).

In summary, protective autophagy and the tumor-associated microbiota-mediated HIF-1α/mitophagy axis, along with the LPS/ER stress axis, together form the core mechanisms underlying radioresistance in solid tumors such as NSCLC. Combining autophagy inhibition with the blockade of microbial signaling represents a key strategy to overcome radioresistance. The LPS/ER stress–autophagy and HIF-1α/mitophagy axes are supported by causal mechanism evidence (category ii) established largely in non-irradiated or non-tumor systems and are extrapolated to the radioresistance setting (category iii).

## 7 Maintenance of cancer stem cell stemness

CSCs contribute significantly to tumor recurrence after therapy due to their self-renewal and differentiation capacities, as well as their resistance to radiotherapy and chemotherapy. They are believed to originate either from mutations in normal stem cells or from the dedifferentiation of cancer cells, and they differ substantially from normal stem cells in terms of their biological behavior and regulatory mechanisms (102). CSC stemness is highly dependent on intrinsic signaling pathways, including Notch, Wnt, Hedgehog, and PI3K/Akt/mTOR, which regulate self-renewal, suppression of differentiation, therapeutic resistance, and tumor progression through distinct molecular mechanisms. Extensive crosstalk among these pathways forms an integrated regulatory network that constitutes the core framework sustaining CSC stemness (103). Although direct evidence that intratumoral microbes maintain CSC stemness is currently lacking, substantial indirect findings suggest that these microbes may influence the CSC niche by modulating key signaling pathways.

*Fusobacterium nucleatum(*Fn*)*, which is abundant in colorectal cancer tissues, has been identified as a key microbial factor that disrupts epithelial homeostasis and enhances the functional properties of tumor cells with CSC-like characteristics. Fn engages its surface adhesin, predominantly FadA (*Fusobacterium* adhesin A), to bind with high affinity to E-cadherin (epithelial cadherin) on host epithelial cells. This interaction has dual mechanistic consequences. On the one hand, FadA-mediated engagement directly disrupts epithelial adherens junction integrity by inducing E-cadherin phosphorylation and promoting clathrin-dependent endocytosis. On the other hand, it initiates the nuclear translocation of β-catenin. In parallel, Fn–FadA signaling perturbs the β-catenin phosphorylation–ubiquitination–degradation axis. Although β-catenin is normally earmarked for ubiquitin-dependent proteolysis following phosphorylation, this pathway reduces β-catenin phosphorylation, suppresses its ubiquitination, and thereby facilitates its cytoplasmic accumulation and subsequent nuclear entry. Once it has translocated into the nucleus, β-catenin drives sustained activation of the Wnt/β-catenin signaling cascade, leading to the upregulation of downstream targets such as MYC (MYC proto-oncogene, which regulates proliferation and stemness features) and CCND1 (cyclin D1,which promotes cell cycle progression). Through these coordinated mechanisms, Fn markedly enhances the self-renewal capacity—a hallmark functional trait—of colorectal cancer stem-like cells, expands the subpopulation with tumor-initiating potential, and ultimately potentiates the malignant growth characteristics of colorectal tumors (104, 105). Concurrently, Fn-derived lipopolysaccharides and other MAMPs activate the TLR4–MyD88–NF-κB signaling cascade, leading to the upregulation of proinflammatory cytokines such as IL-6, IL-8, and TNF-α. These inflammatory cues functionally cooperate with the Wnt pathway to sustain CSC traits and antiapoptotic phenotypes. Although pairwise interactions among Fn infection, inflammatory responses, and tumor stemness (CSC properties) have been well documented, whether these processes form a “self-reinforcing” regulatory loop specifically under Fn-driven conditions remains unclear. The precise molecular nodes that might interconnect and amplify these pathways have yet to be directly validated experimentally (106, 107).At the metabolic level, F. nucleatum has been shown to promote glycolytic reprogramming through an ENO1-IT1-dependent mechanism in colorectal cancer models (108). However, this study did not directly evaluate radiotherapy response. In clinical cohorts, higher intratumoral F. nucleatum abundance has been associated with poorer colorectal cancer-specific survival (109). These findings provide mechanistic and clinical evidence for the biological and prognostic relevance of F. nucleatum, respectively, but do not by themselves establish a direct causal role in radiotherapy resistance. Clinical evidence clearly shows that a high intratumoral abundance of Fn is strongly associated with a poor prognosis, chemotherapy resistance, and an increased risk of recurrence (107, 109). Notably, Fn–FadA signaling drives β-catenin activation and sustains CSC stemness features (104, 105).In preclinical models, eradication of Fn with metronidazole has been shown to restore sensitivity to chemoradiotherapy (110, 111); however, its therapeutic efficacy in humans remains under clinical evaluation. Taken together, current evidence supports Fn as a regulator of CSC-like traits through FadA–E-cadherin–β-catenin/Wnt signaling, TLR4–MyD88–NF-κB inflammatory signaling, and ENO1-IT1/ANGPTL4-associated metabolic reprogramming (causal mechanism evidence, category ii) (103–108), with clinical associations supporting prognostic relevance (category iii) (108, 109). Antibiotic-associated reduction of oral/tumor microbiota, including Fn, has been reported to improve radiotherapy response in colorectal cancer mouse models (111); however, because metronidazole is not Fn-specific and single-microbe depletion/reconstitution with irradiation has not been performed, the Fn-specific causal attribution to radioresistance should be regarded as category (ii), while the broader “microbiota depletion plus irradiation” observation is direct for depletion overall but not taxon-specific. The precise molecular mechanisms linking Fn-maintained CSC stemness to radioresistance therefore remain to be validated in irradiated tumor models.

## 8 Conclusions and perspectives

The intratumoral microbiota profoundly regulate tumor radiosensitivity through multidimensional and bidirectional mechanisms, exerting effects that range from pronounced radiosensitization to the induction of refractory radioresistance. Overall, these effects involve primarily pathways related to DNA damage and repair, ROS generation and oxidative stress, metabolic reprogramming, remodeling of the tumor immune microenvironment, and the regulation of the cell cycle, autophagy, hypoxia, and cancer stem cell maintenance (**Figure 1**). These mechanisms do not operate in isolation; rather, they are highly interconnected and synergistic, ultimately determining whether radiation-induced DNA damage can be converted into irreversible cell death.

Specifically, certain microbial genotoxins or tumor-associated microbes are candidate radiosensitizers. Colibactin-producing pks+ E. coli and CDT-producing bacteria can directly increase DNA lesions; S. aureus and enterotoxigenic B. fragilis can increase ROS-associated DNA damage; HR-HPV, C. trachomatis, and H. pylori can perturb HR or repair-pathway choice; and Leptotrichia enrichment has been associated with favorable outcomes in head and neck cancer. With the exception of defined microbial products combined with irradiation in the same experimental system, these mechanisms should be considered candidate radiosensitizing pathways rather than established clinical radiosensitizers. In contrast, several microorganisms or microbial programs have been proposed to attenuate radiotherapy efficacy, including Fusobacterium nucleatum, tumor-resident Lactobacillus iners–derived lactate, Malassezia-associated complement/MDSC recruitment, butyrate/SCFA-dependent immune and DDR modulation, hypoxia-aggravating facultative anaerobes, LPS/ER-stress–autophagy signaling, and CSC-supporting pathways. However, the strength of evidence is heterogeneous: L. iners–lactate and C. novyi-NT bacteriolytic therapy currently provide direct experimental evidence in irradiated systems, whereas most other mechanisms remain causal mechanism evidence or indirect/extrapolated evidence requiring tumor-site-specific validation.

In summary, the effects of intratumoral microorganisms on tumor radiosensitivity are critical and highly complex, providing new perspectives for understanding radioresistance. Future efforts integrating metagenomics and spatial transcriptomics may enable the precise identification of functional microbial taxa, while therapeutic modulation through antibiotics, antifungals, probiotics, or engineered microbes may improve radiotherapy responses and delay resistance in selected patients. Although this field remains in an early stage, accumulating evidence highlights its potential clinical value as an adjunct to radiotherapy, warranting deeper investigation and validation.

**Table 1** systematically maps these mechanisms to their evidence categories and key references.

**Table 1:** Systematic mapping of levels of evidence to proposed mechanisms underlying intratumoral microbiota-mediated modulation of radiosensitivity.

Evidence category	Proposed mechanism (representative microbes)	Key references
(i) Direct experimental evidence	L. iners-derived L-lactate stabilizes HIF-1α, sustains glycolysis and ATP generation, accelerates γ-H2AX clearance, and confers chemoradioresistance in cervical cancer cell lines and patient-derived organoids.	(79)
(i) Direct experimental evidence	C. novyi-NT germinates in hypoxic tumor regions and lyses hypoxic tumor cells, enhancing the efficacy of radiotherapy (69); its additional immunostimulatory effects have been reported separately (70).	(69, 70)
(ii) Causal mechanism evidence	Antibiotic-mediated depletion of oral/tumor-associated microbiota, including F. nucleatum, improves radiotherapy response in colorectal cancer models; the Fn-specific contribution remains unresolved.	(111)
(ii) Causal mechanism evidence	pks+ E. coli colibactin alkylates DNA, generating interstrand crosslinks that are converted to double-strand breaks during repair.	(23, 24)
(ii) Causal mechanism evidence	Cytolethal distending toxin (C. jejuni, S. Typhi, H. ducreyi) CdtB acts as a DNase I-like nuclease to induce DNA strand breaks.	(25–27)
(ii) Causal mechanism evidence	S. aureus induces ROS/RNS, γ-H2AX and 8-OHdG lesions; ETBF BFT upregulates spermine oxidase (SMO) to generate ROS and activate the NLRP3 inflammasome, damaging host-cell DNA.	(29–32)
(ii) Causal mechanism evidence	F. nucleatum FadA engages E-cadherin and activates Wnt/β-catenin signaling, increasing MYC and CCND1 expression and promoting CSC-like properties. Additional F. nucleatum-associated metabolic and inflammatory pathways may support tumor-cell survival, but their contribution to CSC maintenance and radiotherapy resistance remains inferential.	(104–106, 108)
(ii)/(iii) Causal mechanism / extrapolated evidence	HR-HPV oncogenes, C. trachomatis (PP2A–Chk2) and H. pylori (SNHG17) impair HR or bias repair toward error-prone NHEJ; these mechanisms are established largely in non-irradiated or extra-tumoral systems (category ii), and their translation to intratumoral radiation response currently represents extrapolated evidence (category iii).	(36–38)
(ii)/(iii) Causal mechanism / extrapolated evidence	Chlamydia trachomatis disrupts PP2A signaling and activates Chk2, inducing G2/M arrest; because G2/M-phase cells are highly radiosensitive, this mechanism may enhance radiation sensitivity, although direct RT evidence is lacking.	(37, 43–45)
(ii)/(iii) Causal mechanism / extrapolated evidence	F. nucleatum activates ROS-generating and TLR4–NF-κB signaling and has been associated with oxidative DNA damage and impaired MMR; the relevance of this axis to radioresistance remains inferential.	(41, 42)
(ii) Causal mechanism evidence	P. anaerobius engages integrin α2/β1–FAK and activates PI3K/Akt–GSK3β and NF-κB, stabilizing cyclin D1 and driving G1/S transition (general cyclin-D1 control in ).	(46–49)
(ii)/(iii) Causal mechanism / extrapolated evidence	Innate immune pathways including cGAS–STING, NLRP3, and TLR4–MyD88–NF-κB may provide mechanistic links between microbial sensing and antitumor immunity; however, direct evidence for these pathways in intratumoral bacteria during radiotherapy remains limited.	(65, 67, 68)
(ii)/(iii) Causal mechanism / extrapolated evidence	Malassezia activates the MBL complement pathway and promotes MDSC recruitment in pancreatic cancer (71, 72). Butyrate/SCFA-mediated immune suppression has been reported mainly in gut-microbiota studies and may be relevant to the tumor setting, but its role in radiotherapy remains extrapolative (14, 73–75).	(14, 71–75)
(ii) Causal mechanism evidence	S. Typhimurium downregulates VEGF-A via AKT/HIF-1α and impairs endothelial cells, suppressing tumor angiogenesis.	(91, 92)
(ii) Causal mechanism evidence	Facultative anaerobic bacteria may contribute to hypoxia-associated mitophagy through HIF-1α–BNIP3/NIX signaling; however, direct evidence linking intratumoral bacteria to this pathway and radiotherapy resistance remains limited (89, 98, 99).	(89, 98, 99)
(iii) Indirect or extrapolated evidence	KSHV (HIF-1α, PI3K/AKT/mTOR) and HCMV (glycolysis, TCA cycle, fatty-acid synthesis) reprogram infected-cell metabolism; established in non-tumor cells without a radiation endpoint, so a radioresistance effect is extrapolated.	(80, 82, 83)
(ii)/(iii) Causal mechanism / extrapolated evidence	Facultative anaerobes upregulate glucose transporters, increasing glucose uptake and accelerating lactate production; effects extrapolated from gut-microbiota and non-radiotherapy systems.	(81)
(ii)/(iii) Causal mechanism / extrapolated evidence	FAO supports PARP1-dependent DSB repair in a non-microbial tumor-cell system (ii); the association of intratumoral taxa (Desulfovibrio, Desulfotomaculum, Streptococcus, Mycobacterium) with fatty-acid metabolism rests on TCGA correlation (iii).	(84, 85)
(iii) Indirect or extrapolated evidence	Intratumoral LPS derived from Escherichia–Shigella and Enterobacteriaceae promotes NSCLC proliferation through the TLR4–mTOR–NF-κB–IL-6 axis; its contribution to autophagy-mediated radioresistance remains unestablished.	(100)
(iii) Indirect or extrapolated evidence	Gut-microbiota-derived butyrate/SCFAs inhibit HDACs and modulate DNA-repair gene expression; effects extrapolated from the gut to the intratumoral setting.	(34, 35)
(iii) Indirect or extrapolated evidence	Gut microbiota–immune axes (L. reuteri tryptophan/AhR metabolites; butyrate-induced M2 polarization) modulate immunotherapy response and are extrapolated to radiotherapy.	(13, 14)
(iii) Indirect or extrapolated evidence	TCGA-based analyses associate intratumoral microbial composition with fatty-acid metabolism and immune dysfunction (TIDE scores); correlative rather than causal.	(84)
(iii) Indirect or extrapolated evidence	Clinical associations: intratumoral Leptotrichia correlates with CD8+ T-cell/M1 infiltration and favorable outcome in head and neck cancer (66); high F. nucleatum abundance correlates with poor prognosis and chemoresistance (107, 109).	(66, 107, 109)
(iii) Indirect or extrapolated evidence	AvrA-mediated β-catenin/Wnt stabilization, KSHV/HCMV metabolic effects (80, 82, 83), and microbial maintenance of CSC stemness (103–107) are plausible routes to radioresistance that remain hypotheses awaiting direct validation in irradiated tumor models.	(50, 51, 80, 82, 83, 103–107)

The evidence supporting these mechanisms is heterogeneous, and we explicitly distinguish direct experimental evidence (category i), causal mechanism evidence (category ii), and indirect or extrapolated evidence (category iii), as detailed in Section 1.2 and summarized in **Table 1**. Direct experimental evidence that tumor-resident microbes alter radiation response in the same tumor system remains scarce and is currently limited to a few examples, including L. iners-derived lactate and C. novyi-NT bacteriolytic therapy; metronidazole-mediated eradication of tumor-resident F. nucleatum currently provides causal mechanism evidence (category ii) that awaits confirmation in additional irradiated tumor models (111). Many mechanistic claims—such as microbial genotoxin-induced DNA damage, Fn-driven Wnt/β-catenin activation, innate immune pathway modulation, and hypoxia-aggravating effects—are supported by causal mechanism evidence established largely outside the irradiation context, whereas several proposed mechanisms, notably Salmonella AvrA-mediated cell-cycle modulation, viral metabolic reprogramming, and microbial maintenance of CSC stemness, currently rest only on indirect or extrapolated evidence. Future studies should therefore prioritize gnotobiotic and antibiotic-depletion models combined with irradiation, intratumoral microbial reconstitution experiments, and longitudinal clinical sampling with radiotherapy endpoints, so as to upgrade the evidence for these mechanisms from category (ii) or (iii) to category (i) and to inform microbiota-targeted radiosensitization strategies.
